# Soil Bacterial Communities Respond to Mowing and Nutrient Addition in a Steppe Ecosystem

**DOI:** 10.1371/journal.pone.0084210

**Published:** 2013-12-31

**Authors:** Ximei Zhang, Quansheng Chen, Xingguo Han

**Affiliations:** 1 State Key Laboratory of Forest and Soil Ecology, Institute of Applied Ecology, Chinese Academy of Sciences, Shenyang, China; 2 State Key Laboratory of Vegetation and Environmental Change, Institute of Botany, Chinese Academy of Sciences, Beijing, China; Charité-University Medicine Berlin, Germany

## Abstract

In many grassland ecosystems, nitrogen (N) and phosphorus (P) are added to improve plant productivity, and the aboveground plant biomass is mowed and stored as hay for the bullamacow. Nutrient addition and mowing affect the biodiversity and ecosystem functioning, and most of the previous studies have primarily focused on their effects on macro-organisms, neglecting the responses of soil microbial communities. In this study, we examined the changes in three community attributes (abundance, richness, and composition) of the entire bacterial kingdom and 16 dominant bacterial phyla/classes in response to mowing, N addition, P addition, and their combinations, by conducting a 5-year experiment in a steppe ecosystem in Inner Mongolia, China. Overall, N addition had a greater effect than mowing and P addition on most of these bacterial groups, as indicated by changes in the abundance, richness and composition in response to these treatments. N addition affected these soil bacterial groups primarily through reducing soil pH and increasing available N content. Meanwhile, the 16 bacterial phyla/classes responded differentially to these experimental treatments, with Acidobacteria, Acidimicrobidae, Deltaproteobacteria, and Gammaproteobacteria being the most sensitive. The changes in the abundance, richness, and composition of various bacterial groups could imply some potential shift in their ecosystem functions. Furthermore, the important role of decreased soil pH caused by N addition in affecting soil bacterial communities suggests the importance of restoring acidified soil to maintain soil bacterial diversity.

## Introduction

With the growth of human population and improvement in the living standard, there is an urgent demand for the development of livestock husbandry. The traditional strategy used for the development of graziery is to increase the bullamacow number; however, this has led to the degeneration of grassland ecosystems [1]–[4]. To reconcile the conflict between graziery development and ecosystem conservation, it is suggested that specific methods (such as addition of nitrogen (N) and phosphorus (P) and mowing) be adopted to improve forage grass productivity in a small area of grassland and leave a large part of the grassland area for maintaining its other ecosystem functions [5], [6]. Mowing and nutrient addition have profound influences on the biodiversity and ecosystem functioning, and previous studies have mainly focused on their influence on higher organisms [5]–[7]. Although soil microbial communities are among the most abundant and diverse groups of organisms on Earth and are responsible for numerous key ecosystem processes [8]–[10], their response to these treatments and, especially, their combinations, has not been comprehensively explored.

Because anthropogenic N deposition is one of the major environmental changes that have greatly altered the processes, functions and services of various terrestrial ecosystems [11], its influence on soil microbial communities and their associated functions has received much attention in the past few decades. Actually, only the response of some microbial functional groups (e.g. the ammonia-oxidizing bacteria and denitrifying bacteria) or the entire soil microbial (or bacterial) community as a whole was often investigated [12], [13]. In particular, the changes in some community attributes (e.g. abundance, diversity and composition), enzyme activities and functional indices (e.g. N cycling rates) were often detected, and these changes were even found to be interrelated sometimes [14]. For example, it was found that in temperate hardwood and pine forests, chronic N deposition reduced the bacterial/fungal biomass ratio and the activity of a fungal lignin-degrading enzyme, and it might be these reductions that caused the decreased litter decomposition rates and altered N cycling rates [15]. Soil microbial communities comprise diverse taxonomic groups (e.g. different phyla/classes) with different phylogenetic histories, physiological traits and ecological functions; however, the response of various taxonomic groups to the environmental changes has not been investigated comprehensively due to the technological limitation [16]. For example, the traditional Sanger DNA sequencing technology was too labor-intensive for the simultaneous measurement of community structure of various bacterial phyla/classes. Fortunately, the current metagenomic approaches that exploit next-generation sequencing technologies (such as the pyrosequencing) can acquire millions or even billions of DNA sequences in only a run, thus enabling the investigation of the diversity and composition of various bacterial phyla/classes rapidly, accurately and simultaneously [17], [18].

Carbon (C), N and P are the most basic compositional elements of an organism. Logically, mowing removes much plant biomass from the ecosystem, and the main effect of mowing on soil microbial communities is the reduction in the amount of C resource (or energy resource) supplied to soil microorganisms [19], [20]. In contrast, N and P addition increases the content of soil nutrients supplied to the soil microbes. Thus, these human activities will likely affect the abundance, richness, and composition of soil microbial communities and various microbial taxonomic groups may respond differently. Meanwhile, a relatively stable C/N/P ratio exists in the elemental composition of a given soil microbial taxonomic group [21], [22]. Because mowing, N and P addition will change the stoichiometric ratios (including C/N, C/P and N/P) of these elements in the soil, these changes will also affect soil microbial communities. In addition, these human activities may even have interactive effects on various microbial communities. For example, P addition was found to only affect the abundance of soil ammonia-oxidizing bacteria in the presence of added N, and not in the absence of N addition [23]. Furthermore, besides the contents of soil C, N and P, these human activities may change other soil physicochemical factors, which will also lead to the alteration in soil microbial communities. For example, it was found that N addition not only leads to increased soil total N content, but also changes in the NH_4_^+^-N content, NO_3_^−^-N content and pH, as well as changes in the heterogeneity of the four indices [24]. And N addition also leads to the changes in the biomass, diversity and composition of the plant communities. All these changes may further lead to the alteration in the soil microbial abundance, diversity and composition [24]. It is worth noting that soil pH has been found to be the most important ecological factor determining soil bacterial community structure [18], [25], suggesting that the changes in soil pH may be the primary route through which N addition alters the bacterial community. Overall, there is an urgent need to study the effects of mowing, N addition, P addition and their combinations on soil microbial communities and to elucidate the underlying mechanisms.

The semiarid temperate steppe in northern China is an important part of the Eurasian grassland biome. Adoption of treatments such as mowing and nutrient addition has been suggested to improve plant productivity in this area [5], [6]. To comprehensively examine the response of soil microbial communities to these managements in the steppe ecosystem, a field manipulative experiment of mowing, N addition, and P addition with eight treatments (control, mowing, N addition, P addition, mowing and N addition, mowing and P addition, N and P addition, and simultaneous mowing, N addition and P addition) was conducted from 2005. The pyrosequence technology targeting bacterial 16S rRNA gene was used to measure the structure of soil bacterial communities [17]. The specific questions of this study were as follows: (1) Whether and how the abundance, richness, and composition of the entire soil bacterial kingdom and the dominant phyla/classes get affected by mowing, N addition, P addition, and their combinations? (2) What are the ecological mechanisms of these treatments altering soil microbial communities?

## Materials and Methods

### Study site and experimental design

This study was part of a long-term experiment conducted at the Duolun Restoration Ecology Station of Institute of Botany, Chinese Academy of Sciences, approximately 30 km from Duolun County (42°02′N, 116°17′E), Inner Mongolia Autonomous Region of China. Our field studies did not involve endangered or protected species, so no specific permissions were required for the location/activity. The experimental site was a typical temperate zone characterized by a semiarid continental monsoon climate. Mean annual temperature was 2.1°C with monthly mean temperature ranging from 18.9°C in July to −17.5°C in January. Mean annual precipitation was about 385.5 mm with 80% precipitation occurred from June to September. Soil was chestnut soil (Chinese classification), corresponding to Calcis-orthic Aridisol in the US Soil Taxonomy classification, with sand, silt, and clay being 62.7%, 20.3%, and 17.0%, respectively [26], [27]. Mean soil bulk density was 1.31 g/cm^3^. This temperate steppe was dominated by perennials, including *Stipa krylovii*, *Artemisia frigida*, *Potentilla acaulis*, *Cleistogenes squarrosa*, *Allium bidentatum*, and *Agropyron cristatum*.

This experiment commenced from 2005. The effects of mowing, N, P, and their combinations were investigated using a nested design with mowing as the primary factor and nutrient addition as the secondary factor. There were four replicates for each of the eight treatments. Within a 199 m×265 m area, eight 60 m×92 m primary plots were set-up with a 5-m-wide buffer zone among plots. Four primary plots were randomly assigned to the mowing treatment and the other four were controls. Aboveground plants were mowed on 20 August every year, leaving only 10 cm of stubble. Within each of the eight primary plots, four 28 m×44 m secondary plots were set-up, with a 1-m buffer zone between them. Each of the four secondary plots was randomly assigned to N addition, P addition, simultaneous N and P addition, and control treatments. N was added in the form of urea in 2005 and NH_4_NO_3_ in 2006–2010 at a rate of 10 g N m^−2^ y^−1^. P was added in the form of calcium superphosphate at a rate of 5 g PO_4_^−3^ m^−2^ y^−1^. Both N and P were added on a rainy day in the middle of July every year.

### Sampling and measurement of bacterial abundance and community structure

Soil samples were taken on 22 August of 2010. Four soil cores (10 cm deep, 3.5 cm diameter) were collected from each secondary plot at random and thoroughly mixed. Soil total C (TC) content was quantified with the potassium dichromate–vitriol oxidization method [28]. Soil total N (TN) content was measured using an Alpkem autoanalyzer (Kjektec System 1026 Distilling Unit, Sweden) according to the Kjeldahl acid–digestion method [28]. Soil total P concentrations (TP; % of dry mass) were measured by the ammonium molybdate method after persulfate oxidation [28]. Soil available N content was determined on a FIAstar 5000 Analyzer (Foss Tecator, Denmark) after extraction of fresh soil with 1 mol/L KCl. Soil pH was measured in 1∶2.5 (W/V) suspensions of soil in distilled water. Soil water content was determined as the weight loss after drying for 24 h at 105°C. For each soil sample, we extracted DNA from 0.5 g of mixed soil using the Fast DNA SPIN kit for soil according to the manufacturer's instructions (Qbiogene, Carlsbad, CA, USA); however, we used 350 µL instead of 50 µL DNA elution solution to elute the DNA in the tenth step of the procedure. Purity and quantity of DNA were checked using a Nanodrop® ND-1000 UV–vis Spectrophotometer (Thermo, USA). The DNA solution was then stored at −20°C until analysis.

To estimate the abundance of the entire bacterial community, the content of bacterial 16S rRNA gene was measured using real-time PCR according to a procedure similar to that described by Fierer et al. [16]. The standard template was a plasmid containing a copy of the *Escherichia coli* 16S rRNA gene, and the standard curve was generated using a 10-fold serial dilution of the template across five orders of magnitudes (2.73×10^4^∼2.73×10^8^ copies). The 20 µL PCR reaction mixtures contained 10 µL SYBR Premix (2×) (TaKaRa Biotechnology Co., Ltd., China), 0.4 µL each of 10 µmol/L forward and reverse primers (Eub338: 5′-ACT CCT ACG GGA GGC AGC AG-3′; Eub518: 5′-ATT ACC GCG GCT GCT GG-3′), 0.4 µL Rox II, 2 µL BSA (10 mg/mL), and 5.8 µL sterile and DNA-free water. The amount of standard and soil DNA samples added per reaction was 1.0 µL (1.2–5.1 ng). The reaction was conducted with a Roche LightCycler™ Real-time PCR system using the following program: 95°C for 1 min followed by 40 cycles of 95°C for 5 sec, 55°C for 15 sec and 72°C for 15 sec. The melting curve analysis was performed to confirm PCR product specificity after amplification by measuring fluorescence continuously as the temperature increased from 65°C to 95°C. Gel electrophoresis analyses were also conducted to confirm that the amplified products were of the appropriate size. The equation Eff = [10^(−1/slope)^−1] was used to calculated the amplification efficiencies and resulted in the value of 97%. The bacterial 16S rRNA gene copy number was calculated using a regression equation that related the cycle threshold (Ct) value to the known number of copies in the standards. Three no-template controls were run for each quantitative PCR assay. For each soil sample, the qPCR reactions were repeated three times. We added BSA to the PCR reaction mixtures to reduce the inhibitory effects of co-extracted polyphenolic compounds in the soil. Additionally, three rounds of PCR were conducted after adding known amounts of standard plasmid with the soil DNA extract to estimate the possible inhibitory effects of co-extracted polyphenolic compounds. The inhibitory effects were found to be negligible.

The method of 454 pyrosequences was used to measure the bacterial community structure of each soil sample. The primers 27F (5′-AGA GTT TGA TCC TGG CTC AG-3′) and 338R (5′-TGC TGC CTC CCG TAG GAG T-3′) were used to amplify the fragment of 16S rRNA gene. To measure all 32 samples in a run, a unique 10-mer tag for each sample was added to the 5′-end of the primer 338R [17]. Each 20 µl PCR mixture contained 4 µl FastPfu Buffer (5×; Transgen), 2 µl of 2.5 mM dNTPs, 0.4 µl of each primer (5 µM), 0.8 µl of DNA template, and 0.4 µl of FastPfu Polymerase (Transgen). The PCR protocol was as follows: 95°C for 2 min (denature); 25 cycles of 95°C for 30 sec (denature), 55°C for 30 sec (anneal), 72°C for 30 sec (elongate); and 72°C for 5 min (elongate). Three replicates of PCR were performed for each sample, after which the products were combined and purified by agarose gel electrophoresis, recovered, and quantified with PicoGreen using a TBS-380 Mini-Fluorometer. Equal molar concentrations of PCR products for each sample were then pooled and sequenced in a Roche 454 Genome Sequencer FLX Titanium system at Shanghai Majorbio Bio-pharm Technology Co., Ltd. The sequence reads for all samples have been deposited in the National Center for Biotechnology Information Sequence Reads Archive (accession no. SRA057669).

### Data analysis

The pyrosequence reads were analyzed mainly with the Mothur software (Version 1.19) [29]. Briefly, the raw reads were first assigned to samples according to their tags and then the standard primers and barcodes were trimmed off, after which reads with length less than 150 bp or with ambiguous characters were removed. The chimeric sequences were also excluded by the chimera.uchime command with default parameters. While the existence of pyronoise may lead to the overestimation of bacterial OTU richness, the removal of pyronoise may remove some actual but rare sequences and further result in the underestimation of OTU richness. Therefore, the step of pyronoise removal was not adopted in this study, and the influence of pyronoise was taken as a systematic error. The V3 region of 16S rRNA gene of the remaining reads were aligned to the Silva database (Version 106) to determine their taxonomic classification and non-bacterial reads were further removed [30]. To minimize the influence of unequal sampling on the following calculated indices, we randomly selected 3,478 reads for each sample. All these sequences (3,478×32) were clustered into OTUs (operational taxonomic units) with larger than 97% similarity, and the consensus taxonomy for an OTU was determined using classify.otu command with confidence threshold 80%. The OTU number of 3,478 reads for each sample was used to represent the OTU richness of the entire bacterial community. For each pair of samples, the Bray-Curtis distance basing on OTU abundance was calculated to represent the compositional variation of the entire bacterial community [31]. Briefly, we first calculated the difference in the number of reads for each OTU within this pair of samples, and then calculated the sum of the absolute values of these differences for all OTUs. Finally, we divided the sum by 6,956 (3,478×2) to represent the Bray-Curtis distance.

For each of the 16 dominant bacterial phyla/classes, (its sequence number in all 3,478 sequences)/3,478 was calculated to represent its relative abundance. To compare the OTU richness of each phylum/class among different samples, we used a different calculation approach from that for the entire bacterial community, because there was different number of sequences for the same phylum/class among different samples. For each phylum/class, we randomly selected a certain number of representative sequences from a sample and calculated the OTU number represented by these sequences, repeated this process 1,000 times and used the mean OTU number to represent its richness. For each of the 16 phyla/classes, we also calculated the Bray-Curtis distance basing on the abundance of OTUs to represent the compositional variation between each pair of samples [31].

Three-way analysis of variance (ANOVA) was used to determine the main and interactive effects of mowing, N addition and P addition on the nine soil physicochemical indices, including soil TC content, TN content, TP content, the three stoichiometric characteristics (TC/TN, TC/TP, and TN/TP), available N content, pH and water content. Three-way ANOVA was also used to determine the main and interactive effects of mowing, N addition, and P addition on the abundance and OTU richness of the entire bacterial community as well as the relative abundance and OTU richness of each of the 16 dominant phyla/classes. To visualize the compositional variation of the entire bacterial community among different treatments, we used non-metric multidimensional scaling (NMDS) plots with the software of Primer (PRIMER 5 for Windows). Permutational multivariate analysis of variance (PERMANOVA) was further used to reveal the effects of experimental treatments on the composition of the entire bacterial community and that of each of the 16 phyla/classes [32]. Stepwise regression analysis was used to identify the factor that could effectively explain the changes in the abundance, richness, and composition from the aforementioned nine potential soil physicochemical indices. In particular, principal coordinate analyses were first used to determine the difference in community composition among different samples, and the first principal coordinates of the 17 bacterial groups explained 5.01–12.9% of community compositional variation. The first principal coordinate was subsequently used to represent the dependent variable in the stepwise regression analyses. Before regressions, all the data were tested for normal distribution. Collinearity was detected by calculating the condition index for each explanatory variable and it was less than 50 for each variable, suggesting autocorrelation did not occur. Three-way ANOVA and stepwise regression analyses were conducted with SPSS software (SPSS 13.0 for WINDOWS).

## Results

### Soil physicochemical indices

The effects of these experimental treatments on some of the nine soil physicochemical indices have been described previously [23]. Briefly, for all treatments of mowing, N addition, P addition, and their combinations, only N addition significantly affected the three indices of soil TC content, available N content and pH (*P*<0.05; Table S1). Only P addition significantly altered the three indices of soil TP content, TC/TP ratio and TN/TP ratio; while none of the treatments changed the remaining three indices of soil TN content, TC/TN ratio, and water content.

### Bacterial abundance or relative abundance

**Figure 1:**
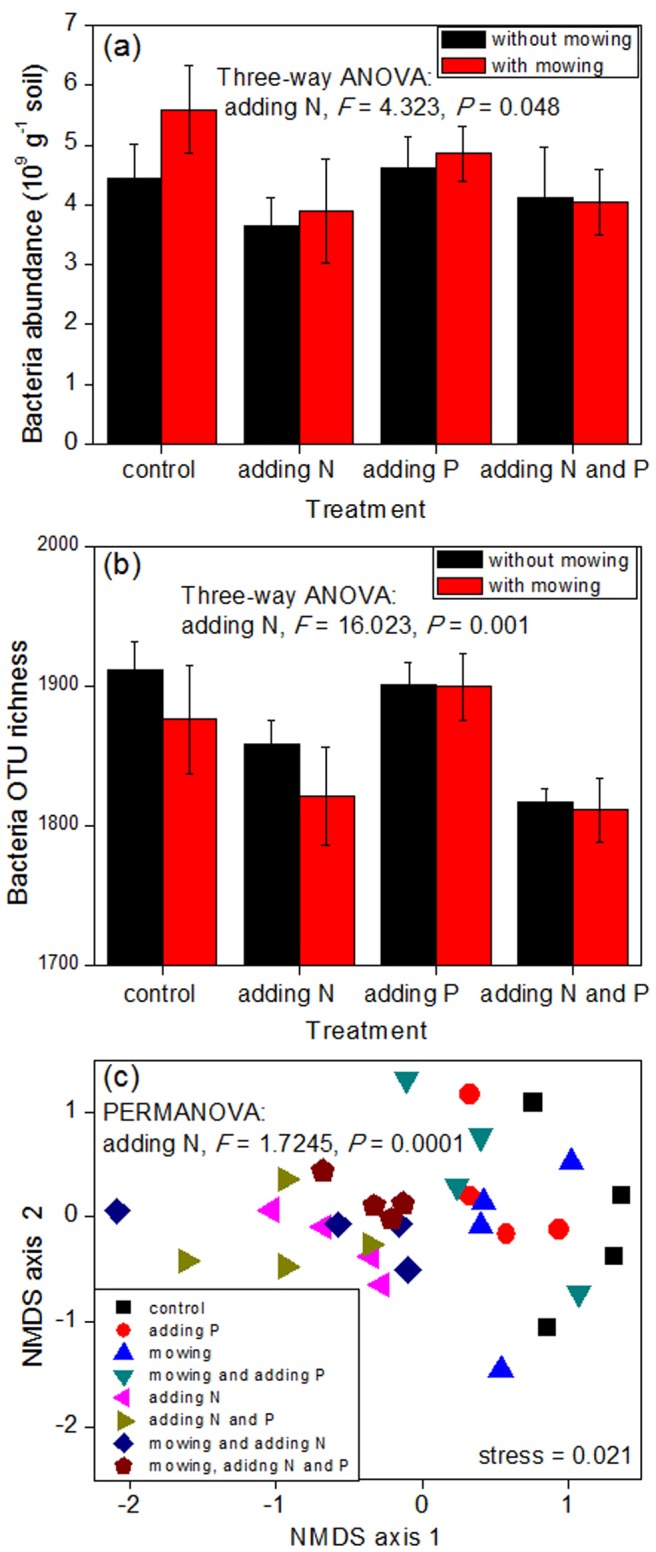
Effects of experimental treatments on the abundance, richness, and composition of the entire bacterial community. For clarity, only the significant statistical results (*P*<0.05) are shown in the figure. In (a) and (b), the bars represent one standard error (n = 4).

**Figure 2:**
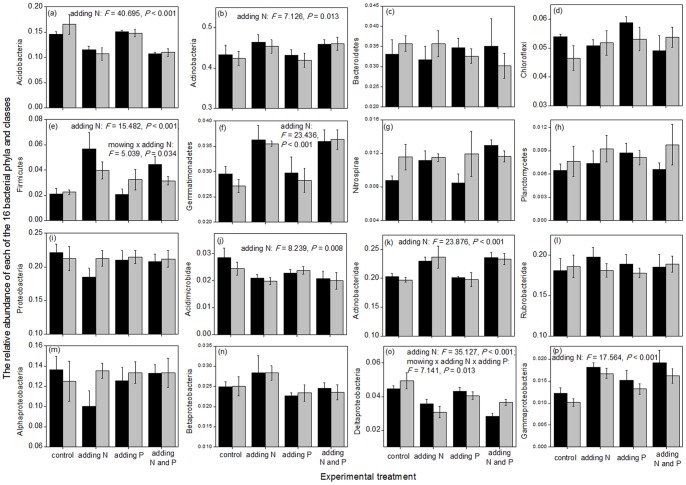
Effects of experimental treatments on the relative abundances of 16 dominant bacterial phyla/classes. Three-way ANOVA was used to test the effect of experimental treatments. For clarity, only the significant statistical results (*P*<0.05) are shown in the figure. The bars represent one standard error (n = 4). The black and gray columns represent the treatments without and with mowing, respectively.

Among the treatments of mowing, N addition, and P addition, only N addition significantly decreased the abundance of the entire soil bacterial community (*P*<0.05; Fig. 1a). Furthermore, the effects of the treatments on the relative abundances of nine dominant bacterial phyla (Acidobacteria, Actinobacteria, Bacteroidetes, Chloroflexi, Firmicutes, Gemmatimonadetes, Nitrospirae, Planctomycetes, and Proteobacteria) and seven dominant classes (Acidimicrobidae, Actinobacteridae, Rubrobacteridae, Alphaproteobacteria, Betaproteobacteria, Deltaproteobacteria, and Gammaproteobacteria) were also examined. N addition significantly decreased the relative abundances of Acidobacteria, Acidimicrobidae, and Deltaproteobacteria, and increased those of Actinobacteria, Firmicutes, Gemmatimonadetes, Actinobacteridae, and Gammaproteobacteria (Fig. 2). Mowing and N addition had an interactive effect on the relative abundance of Firmicutes (Fig. 2e); in particular, although N addition increased the relative abundance of Firmicutes, the extent of increase was less following simultaneous mowing and N addition. Furthermore, mowing, N addition, and P addition also had an interactive effect on the relative abundance of Deltaproteobacteria (Fig. 2o).

### OTU richness

**Figure 3:**
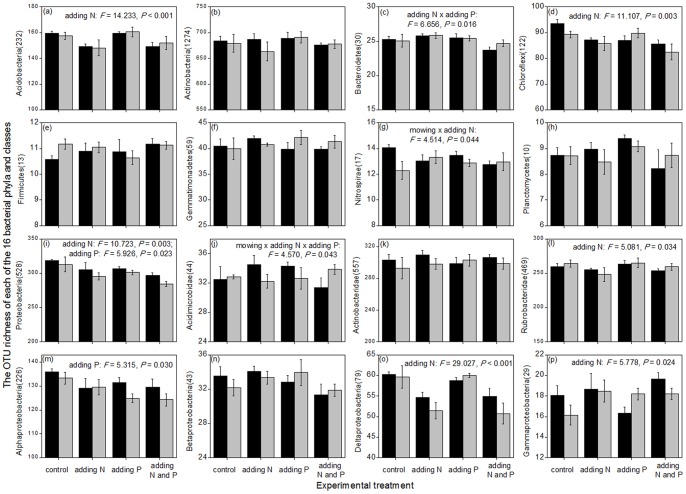
Effects of experimental treatments on the OTU richness of 16 dominant bacterial phyla/classes. Three-way ANOVA was used to test the effect of experimental treatments. For clarity, only the significant statistical results (*P*<0.05) are shown in the figure. The bars represent one standard error (n = 4). The black and gray columns represent the treatments without and with mowing, respectively. The number in the brackets following the phylum/class name (e.g., 232 in Acidobacteria(232) in Fig. 3a) represents the sampled sequence number from which OTU richness was calculated.

Among the treatments of mowing, N addition, and P addition, only N addition significantly decreased the OTU richness of the entire soil bacterial community (*P*<0.05; Fig. 1b). Furthermore, N addition also significantly decreased the OTU richness of Acidobacteria, Chloroflexi, Proteobacteria, Rubrobacteridae, and Deltaproteobacteria, and increased that of Gammaproteobacteria (Fig. 3). On the other hand, P addition significantly decreased the OTU richness of Proteobacteria and Alphaproteobacteria (Fig. 3i, m). N addition and P addition had an interactive effect on the OTU richness of Bacteroidetes (Fig. 3c); in particular, N addition alone and P addition alone had little effect on its OTU richness, but the simultaneous addition of N and P decreased it. Mowing and N addition had an interactive effect on the OTU richness of Nitrospirae (Fig. 3g), whereas mowing, N addition, and P addition had an interactive effect on the OTU richness of Actinobacteridae (Fig. 3j).

### Bacterial composition

Among the treatments of mowing, N addition, and P addition, only N addition significantly altered the composition of the entire soil bacterial communities (*P*<0.05; Fig. 1c). As shown in the NMDS plots in Fig. 1c, the bacterial communities in all the N addition treatments (including N addition, N and P addition, mowing and N addition, and simultaneous mowing, N addition and P addition) shifted relative to those in all the treatments without N addition (including control, P addition, mowing, and mowing and P addition). Furthermore, N addition also significantly altered the composition of each of the 16 bacterial phyla/classes (*P*<0.05; Table 1). Mowing, N addition, and P addition had an interactive effect on Nitrospirae composition, whereas mowing and P addition had an interactive effect on Planctomycetes composition (Table 1).

**Table 1:** The effects of mowing (M), nitrogen addition (N), phosphorus addition (P) and their combination on the composition of the 16 dominant bacterial phyla/classes revealed by PERMANOVA.

Bacterial groups	M		N		P		M×N		M×P		N×P		M×N×P	
	*F*	*P*	*F*	*P*	*F*	*P*	*F*	*P*	*F*	*P*	*F*	*P*	*F*	*P*
Acidobacteria	1.024	0.343	2.188	0.0001*	1.008	0.401	0.961	0.645	0.903	0.879	0.941	0.732	0.937	0.762
Actinobacteria	0.970	0.706	1.433	0.0001*	1.058	0.125	0.972	0.691	1.007	0.407	0.966	0.744	0.986	0.584
Bacteroidetes	1.126	0.135	2.269	0.0001*	1.075	0.222	1.052	0.284	1.107	0.167	0.863	0.896	1.042	0.313
Chloroflexi	1.076	0.165	1.224	0.0063*	1.045	0.277	0.974	0.614	1.108	0.093	1.133	0.052	1.021	0.382
Firmicutes	1.246	0.102	2.010	0.0008*	0.936	0.597	1.299	0.072	0.941	0.578	1.019	0.394	0.836	0.825
Gemmatimonadetes	1.074	0.252	1.556	0.0001*	0.903	0.790	0.822	0.948	1.087	0.216	0.989	0.511	0.982	0.536
Nitrospirae	1.259	0.117	1.738	0.0018*	0.981	0.510	1.260	0.118	1.313	0.079	0.786	0.851	1.533	0.016*
Planctomycetes	0.956	0.650	1.236	0.0193*	1.166	0.067	1.075	0.230	1.194	0.044*	1.070	0.252	0.908	0.805
Proteobacteria	1.012	0.375	2.014	0.0001*	1.077	0.156	0.911	0.885	1.045	0.243	0.910	0.897	1.025	0.320
Acidimicrobidae	1.110	0.174	1.552	0.0001*	0.934	0.718	0.917	0.770	0.934	0.715	0.995	0.514	0.864	0.893
Actinobacteridae	1.024	0.339	1.598	0.0001*	1.101	0.085	1.028	0.317	1.033	0.289	0.938	0.807	0.996	0.490
Rubrobacteridae	0.904	0.941	1.233	0.0004*	1.023	0.346	0.942	0.822	0.931	0.863	1.012	0.417	0.962	0.721
Alphaproteobacteria	1.010	0.402	2.068	0.0001*	1.062	0.248	0.850	0.936	1.057	0.269	0.842	0.952	1.010	0.412
Betaproteobacteria	1.129	0.175	2.283	0.0001*	1.045	0.340	0.874	0.817	1.021	0.405	0.966	0.572	1.009	0.438
Deltaproteobacteria	0.942	0.720	1.674	0.0001*	1.056	0.277	1.017	0.410	0.977	0.579	0.986	0.544	1.065	0.238
Gammaproteobacteria	0.938	0.612	2.307	0.0001*	1.051	0.366	0.956	0.565	1.021	0.422	1.124	0.234	0.744	0.929

The composition of each bacterial phylum/class means the relative abundance of each OTU within this phylum/class. See the effect of these treatments on the composition of the entire bacterial kingdom in Fig. 1c. * denotes *P*<0.05.

### Variables correlated with bacterial abundance, richness, and composition

Among all the 17 bacterial groups (including the entire bacterial kingdom and 16 phyla/classes), stepwise regression analyses revealed that the abundance or relative abundances of 12 groups were explained by the nine measured soil physicochemical factors (Table 2). In particular, the relative abundances of four groups (Bacteria, Firmicutes, Gemmatimonadetes, and Gammaproteobacteria) were correlated with soil pH alone, those of two groups (Actinobacteria and Chloroflexi) correlated with soil available N content alone, and those of two groups (Acidobacteria and Deltaproteobacteria) correlated with both soil pH and available N content. In addition, the relative abundance of Nitrospirae was correlated with TN/TP ratio, that of Acidimicrobidae correlated with both soil pH and TN/TP ratio, that of Actinobacteria correlated with both available N content and TC/TP ratio, and that of Bacteroidetes with soil pH, available N content and water content (Table 2).

**Table 2:** Variables responsible for the changes in abundance, richness, and composition of various bacterial groups.

Index	Bacterial group	Result	r^2^	*P*	effective factors
	Bacteria	y = −4.336*10^9^+1.285*10^9^pH	0.132	0.041	N
	Acidobacteria	y = −0.064−0.001Nav+0.032pH	0.610	<0.001	N
	Actinobacteria	y = 0.414+0.001Nav	0.229	0.006	N
	Bacteroidetes	y = −0.001−0.302water+0.00032Nav+0.007pH	0.463	0.001	
	Chloroflexi	y = 0.057−0.00023Nav	0.171	0.019	
abundance	Firmicutes	y = 0.210−0.026pH	0.302	0.001	N; M×N
	Gemmatimonadetes	y = 0.097−0.010pH	0.647	<0.001	N
	Nitrospirae	y = 0.017−0.001N/P	0.196	0.011	
	Acidimicrobidae	y = −0.021+0.008pH−0.001N/P	0.391	0.001	N
	Actinobacteria	y = 0.152+0.001Nav+0.002C/P	0.608	<0.001	N
	Deltaproteobacteria	y = −0.018+0.009pH−0.00025Nav	0.459	<0.001	N; M×N×P
	Gammaproteobacteria	y = 0.053−0.006pH	0.237	0.005	N
	Bacteria	y = 1913.940−2.348Nav	0.257	0.003	N
	Acidobacteria	y = 163.918−0.428Nav	0.452	<0.001	N
	Bacteroidetes	y = 28.067−7.826P	0.251	0.003	N×P
	Chloroflexi	y = 47.073+5.959pH	0.212	0.008	N
	Firmicutes	y = 13.186−0.899N	0.147	0.030	
richness	Gemmatimonadetes	y = 35.152+1.967C/N	0.199	0.010	
	Planctomycetes	y = 15.814−0.046Nav−0.883pH	0.387	0.001	
	Proteobacteria	y = 161.767+20.720pH	0.254	0.003	N; P
	Alphaproteobacteria	y = 83.713+6.770pH	0.163	0.022	P
	Deltaproteobacteria	y = 61.650−0.241Nav	0.400	<0.001	N
	Gammaproteobacteria	y = 34.431−2.421pH	0.198	0.011	N
	Bacteria	y = 0.944+0.005Nav−0.157pH	0.794	<0.001	N
	Acidobacteria	y = −1.247+0.201pH−0.006Nav	0.719	<0.001	N
	Actinobacteria	y = −0.026−0.007Nav+0.464P	0.594	<0.001	N
	Bacteroidetes	y = −1.424−0.007Nav+0.232pH	0.664	<0.001	N
	Chloroflexi	y = −0.271+0.014C/P	0.295	0.001	N
	Firmicutes	y = 1.957−0.288pH	0.333	0.001	N
	Gemmatimonadetes	y = 0.649−6.484water−0.647P	0.299	0.006	N
composition	Nitrospirae	y = 1.239−0.182pH	0.141	0.034	N; M×N×P
	Proteobacteria	y = 1.048+0.006Nav−0.174pH	0.810	<0.001	N
	Acidimicrobidae	y = −0.553+0.007Nav+0.058N/P	0.489	<0.001	N
	Actinobacteria	y = −0.276+0.006Nav+0.007C/P	0.564	<0.001	N
	Rubrobacteridae	y = 0.086−0.004Nav	0.222	0.007	N
	Alphaproteobacteria	y = 0.888+0.006Nav−0.151pH	0.756	<0.001	N
	Betaproteobacteria	y = 1.025+0.008Nav−0.201pH+0.023N/P	0.801	<0.001	N
	Deltaproteobacteria	y = 1.716−0.252pH	0.374	<0.001	N
	Gammaproteobacteria	y = −1.807+0.266pH	0.382	<0.001	N

Effective factors represent the factors with significant effects on soil bacterial communities. See the details in Figs 1–3 and Table 1. In this part of effective factors, M, N, and P represent mowing, N addition, and P addition, respectively. ‘×’ represents the interaction among different treatments. In the result part, pH, N, P, water, Nav, C/N, C/P, N/P represent soil pH, N content, P content, water content, available N content, C/N ratio, C/P ratio and N/P ratio, respectively. There were 32 samples for the regressions.

Among all the 17 groups, the OTU richness of 11 groups was explained by the soil physicochemical factors (Table 2). Specifically, the richness of four groups (Chloroflexi, Proteobacteria, Alphaproteobacteria, and Gammaproteobacteria) were correlated with soil pH alone, those of three groups (Bacteria, Acidobacteria, and Deltaproteobacteria) correlated with soil available N content alone, and that of one group (Planctomycetes) with both soil pH and available N content. In addition, the richness of Firmicutes, Bacteroidetes, and Gemmatimonadetes was correlated with soil N content, P content, and soil TC/TN ratio, respectively (Table 2).

Among all the 17 bacterial groups, the compositions of 16 groups were explained by the soil physicochemical factors (Table 2). In particular, the compositions of four groups (Firmicutes, Nitrospirae, Deltaproteobacteria, and Gammaproteobacteria) were correlated with soil pH alone, that of one group (Rubrobacteridae) correlated with soil available N content alone, and those of five group (Bacteria, Acidobacteria, Bacteroidetes, Proteobacteria, and Alphaproteobacteria) with both soil pH and available N content. In addition, the composition of Chloroflexi was correlated with soil C/P ratio, that of Actinobacteria correlated with both soil available N content and P content, that of Gemmatimonadetes correlated with both soil P content and water content, that of Acidimicrobidae correlated with both soil available N content and N/P ratio, that of Actinobacteria correlated with both soil available N content and C/P ratio, and that of Betaproteobacteria with all of soil pH, available N content and N/P ratio (Table 2).

## Discussion

Four types of evidence revealed that N addition had a much greater influence on the soil bacterial communities than mowing and P addition. First, with respect to the entire bacterial kingdom, only N addition significantly (*P*<0.05) affected its abundance, richness, and composition, and mowing and P addition had non-significant effects (Fig. 1). Second, with regard to the relative abundances of the 16 bacterial phyla/classes, N addition significantly affected eight groups, whereas mowing affected only two groups (Firmicutes and Deltaproteobacteria) and P addition affected only one group (Deltaproteobacteria; Fig. 2). Third, regarding the OTU richness of the 16 bacterial phyla/classes, N addition significantly affected nine groups, whereas mowing affected only two groups (Nitrospirae and Acidimicrobidae) and P addition affected only four groups (Bacteroidetes, Proteobacteria, Acidimicrobidae, and Alphaproteobacteria; Fig. 3). Finally, with respect to the composition of the 16 bacterial phyla/classes, N addition significantly affected all these groups, whereas mowing as well as P addition, respectively, affected only two groups (Nitrospirae and Planctomycetes) (Table 1).

Mowing had limited influence on soil bacterial communities possibly because it was carried out at the end of the growth season. In other words, the plants would have already supplied much organic C resource (or energy resource) to the soil microorganisms before the mowing time. However, if mowing is performed earlier, then the influence might be much greater. Nevertheless, this hypothesis needs to be tested in future studies. P addition had limited influence on soil bacterial communities, implying that P was not a key limiting element for soil microorganisms in this steppe ecosystem [33].

These experimental treatments affected various soil physicochemical factors (Table S1), which might further lead to changes in the abundance, richness and composition of soil microbial communities. For example, N addition increased soil available N content from 12.912 to 31.369 mg/kg soil (independent of other treatments), which is favorable to soil bacterial communities because N is a limiting element in this steppe ecosystem [33]. However, N addition decreased soil pH from 7.094 to 6.501 (independent of other treatments), which is unfavorable to most of the soil microbial communities because they are most adapted to neutral environments. Stepwise regression analysis revealed the mechanism of these experimental treatments altering soil bacterial communities. For example, the first principal coordinate of the composition of the entire soil bacterial kingdom showed significant correlations with both soil pH and available N content (Table 2). Because soil bacterial composition was demonstrated to be altered significantly only by the treatment of N addition (Fig. 1c), these results suggest that soil pH and available N content are the primary drivers of the change. Overall, there were a total of 39 bacterial groups' attributes correlated with the nine measured soil physicochemical indices (Table 2). In particular, soil pH and available N content were correlated with 23 and 20 bacterial groups' attributes, respectively. All the three stoichiometric indices (TC/TN, TC/TP, and TN/TP) were correlated with eight groups' attributes. Soil TN, TP, and water contents were correlated with one, three, and two groups' attributes, respectively (Table 2). These results suggested that soil pH and available N content affected more bacterial phyla/classes than other measured soil indices.

Terrestrial ecosystems are generally N-limited, so it is traditionally thought that N addition/deposition would favor soil microbial communities by increasing nutrient content [34]–[36]. Although the influence of N addition/deposition on other physicochemical indices (e.g., soil pH and trace elements) has also been widely demonstrated, their contribution to the soil bacterial changes in response to N addition/deposition has not received enough attention [24], [34]. Here our results showed the important role of the decreased soil pH. It is likely because that the intracellular pH of most bacterial species is usually within one pH unit of neutral [25] and the decreased soil pH become a serious selective pressure for them. Actually, a recent study found that the decrease in soil pH caused by N addition was the factor most closely related with the alteration in soil microbial community composition as well as the decline in microbial respiration [37]. Together, these results suggest that some specific strategies (e.g., adding calcium oxide) should be considered to restore soil pH and to maintain soil microbial diversity and their ecosystem functions.

The 16 bacterial phyla/classes examined responded to the experimental treatments with different sensitivities. Acidobacteria, Acidimicrobidae, Deltaproteobacteria, and Gammaproteobacteria were the most sensitive groups, because they exhibited a significant response to the treatments with regard to all the three community attributes of relative abundance, OTU richness, and composition (*P*<0.05; Figs. 2 and 3; Table 1). In contrast, Planctomycetes and Betaproteobacteria were the least sensitive groups, because they responded significantly to the treatments only with respect to the community composition. The remaining 10 groups, including Actinobacteria, Bacteroidetes, Chloroflexi, Firmicutes, Gemmatimonadetes, Nitrospirae, Proteobacteria, Actinobacteridae, Rubrobacteridae, and Alphaproteobacteria, had middling sensitivity, because they responded with respect to two community attributes (Figs. 2 and 3; Table 1).

Soil bacterial communities drive many types of ecosystem functions, and the changes in their abundance, richness, and composition can suggest potential functional shifts. For example, a large proportion of bacteria in the phylum Chloroflexi can acquire energy and fix CO_2_ through photosynthesis [38]–[40]; thus, the decreased OTU richness of Chloroflexi following N addition implies reduction in their C sink function (Fig. 3d). Furthermore, a large proportion of bacteria in the phylum Nitrospirae can transform nitrite into nitrate [41], [42]; thus, the decreased OTU richness of Nitrospirae following mowing (Fig. 3g) suggests that their N-cycling function may be reduced. However, there were no differences in the OTU richness of Nitrospirae following N addition and simultaneous treatment of mowing and N addition, implying that this function was not affected. Many plant pathogenic bacteria belong to the phylum Firmicutes [43]. Although N addition promoted the growth of plant community in the steppe ecosystem [5], [6], it also led to an increase in the relative abundance of Firmicutes (Fig. 2e), implying that the health of the plant community may be threatened. In contrast, mowing decreased plant biomass, and simultaneous mowing and N addition decreased the relative abundance of Firmicutes than N addition alone (Fig. 2e), implying a lower threat to plant health. Nevertheless, as soil bacterial communities are very complex, knowledge about the ecosystem functions of most of the bacterial phyla/classes is still very limited [44], not to mention the functional shifts under the treatments of mowing and nutrient addition.

## Supporting Information

Table S1 **The effect of experimental treatments on nine soil physicochemical indices.**
(XLS)
